# Microscopy of the Teeth

**Published:** 1868-12

**Authors:** S. P. Cutler

**Affiliations:** Recently Professor of Chemistry and Microscopy in the Ohio Dental College, Cincinnati. Formerly Professor of Chemistry and Natural Sciences in the Botanic Medical College, Memphis, Tenn.


### MICROSCOPY OF THE TEETH.

BY S. P. CUTLER, M. D., A. E. G., D. D. S.

Recently Professor of Chemistry and Microscopy in the Ohio Dental College, Cincinnati.
Formerly Professor of Chemistry and Natural Sciences in the
Botanic Medical College, Memphis, Tenn.

### ARSENIOUS ACID IN THE TREATMENT OF DENTAL PULPS.

At the meeting of the Mississippi Valley Dental Association, at the Ohio Dental College on the 4th of March, 1868, the subject of treating pulps with arsenic by Dr. H. A. Smith, was introduced, which led to considerable discussion. In that discussion I maintained that arsenic was taken up by the circulation, sufficient to destroy the pulp. I also maintained, *a priori*, that arsenic was absorbed by the red blood corpuscles, there combining with the iron of the hemætin, formed a poisonous insoluble compound; an arsenuret or something else, from the fact that the hydrated peroxide of iron is the only sure antidote to arsenious acid, when taken into the stomach. From this fact it is but reasonable to conclude that the iron of the blood is in the form of a hydrated peroxide, if in the form of an oxide at all, that being the only soluble oxide in water. I give this as an hypothesis, though a reasonable one. Arsenic does not combine with albumen, neither is albumen an antidote to arsenic, though a most perfect one for corrosive sublimate, which combines chemically, forming an insoluble compound, thereby leading to the conclusion that this last salt in its poisonous effects combined with albumen whereever meeting with it, whether in the tissues or in the blood, combines with a force or affinity sufficiently strong to overcome vital resistance of the tissues. On the other hand arsenic does not readily act on the tissues by forming chemical combinations, as no such combinations can be discovered by microscopic examination of pulps devitalised by that salt, no abnormal appearance save that of the retained blood which has assumed a more livid hue, and a greater or less amount of decomposed corpuscles, their hemætin being darker than normal venous blood. This fact is made more certain and manifest by absorption of this poisonous compound into the tubuli, after the destruction of a pulp by the acid, where the nerve cavity has not been opened to let the blood escape. In such cases the absorbed poisoned hemætin into the crown of a tooth turns it of a dark livid color, which does not happen when spontaneous death of the pulp takes place, nor always when killed with the acid does the change in color of the tooth occur, when neglected as above stated. In the latter case, perhaps, there may not be the same amount of fullness and distension of the blood vessels of the pulp. This absorption and discoloration is the result of arsenious absorption, not ordinary vital absorption as in living tissues. This discoloration of the tooth could not take place from absorption of blood corpuscles, as their diameter is several times as great as the caliber of the tubuli: hence, they could not enter unless broken up. No peculiarity is observed under the microscope from specimens of such teeth, only a slight discoloration scarcely perceptible owing to thinness of specimen. This osmotic absorption must take place with nerve fibrils in teeth in tubuli around them, there being space sufficient, no doubt.

The reason for assuming this last hypothesis is the fact that decomposition of pulp and fibrils has not yet taken place to any great extent, the presence of arsenic retarding to some extent decomposition of animal tissues. Recent researches have shown that a small amount of arsenic is found in the pulp substance when treated with the acid. Corrosive sublimate would perhaps be more effective for the devitalization of pulps than the acid, it being more active and combines more extensively, to wit: with all albumen, nerve tissue, liquor sanguinus, corpuscles and cell tissues of the pulp, chemically uniting, form a poisonous, insoluble chemical compound with all the albumen of the pulp cavity; not so with arsenic, as it does not form any compound with albumen at all, only with the hemætin, notwithstanding Prof. Geo. W. Raines, formerly Professor of Chemistry at West Point, in his *chef d' œuvre* delivered before the Medical Society of Augusta, Ga., when he stated that both arsenic and corrosive sublimate combined with albumen—to the contrary. See *Register*, Sept. '68, p. 382. In cases of poisoning from arsenic, if life has been protracted the mineral, may not be found in the stomach or bowels, but in all of the soft tissues and fluids, especially the glands, as the liver and spleen. *Taylor's Jurisprudence*, p. 80.

The *modus operandi* of arsenic poisoning is, if any odds, more obscure than that of the bichloride of mercury. Taylor's Medical Jurisprudence, also confirmed by Christison and Orfilæ, on poisons states, p. 68, That in some cases of arsenic poisoning, there was no lesion of the mucous membrane of the stomach. In all cases there is inflammation of the mucous membrane of the stomach, and generally of the bowels. In whatever manner the poison is introduced into the system, the symptoms are generally the same; that is, the mucous coat of the stomach become inflamed with vomiting, intense pain, and all the other concomitant symptoms. Ulcerations and perforations are rare of the stomach. The symptoms generally come on from half an hour to an hour after taking a large dose; sometimes in a few minutes, sometimes as late as ten hours, though rare in either extreme. Death from large doses generally takes place between 18 hours and three days, not always governed by the quantity taken, p. 71.

How does arsenic act on the pulp is an important question. Let us examine the subject. When a tooth has decayed to the nerve cavity, the nerve fibrils passing through that portion of dentine removed by decay are carried away more or less, and entirely so when the softened dentine is all removed before introducing the acid. In this condition of the tooth the pulp membrane at the point of decay is perforated by innumerable openings, through which formerly passed the tubular nerve fibrils. These openings furnish free admission for arsenic, if in a state of solution, which takes place no doubt to some extent by the solvent action of the *liquor pulpæ.* If I may be allowed to coin a barbarous word, or the extra pulpal liquor found exterior to the pulp; and so soon as this solution takes place, then it is in an absorbent condition, and may be readily taken into the pulp as fast as it meets with elements for which it has affinities, which agents may be assumed to be in the red corpuscles of the blood in the pulp. From the irritation caused by this poisoned blood, destructive inflammation devitalizes the pulp, in my opinion, by mechanical pressure from the distended vessels, and not from actual poison of the pulp, as the arteries will continue to force blood into the pulp, after the power of the veins to return the blood has ceased. I have assumed this hypothesis from the data furnished by the microscope, which shows no lesion of nerve fibrils or any other signs of lesion whatever, save that of the blood, which does show indications of corpuscular lesion, though my observations have not yet been sufficiently extensive to definitely determine this last point; but in relation to all other lesions of the pulp, I am fully confident in the negative statement above made. Now when this paralyzed pulp is not removed, especially when the paralysis is not complete, in a given time the congested and inflamed pulp may react by the veins and capillaries, gradually recovering their lost *vis a turgo*, and may remove the accumulated and poisoned blood into the general circulation, where it is not felt owing to its insignificancy, beyond at farthest the bottom of the alveolus. Sometimes by puncturing the pulp and letting out the blood freely, after 24 hours the vitality of the pulp returns, after which we find it more difficult to destroy. In some cases a portion sloughs away and may be removed, and the apicial portion reorganizes and retains its sensibility and vitality to an indefinite time, in some cases for years, sometimes with and sometimes without giving trouble. In young subjects it is found frequently more difficult owing to a more open condition of root to destroy the pulp, as the blood vessels have more room to expand and return the blood from the pulp and the poison with it, until it may be all carried from the pulp into the general circulation, that is, all that is taken into and combines with the hemætin of the blood, in consequence leaving the pulp unimpaired to any extent. By reference to a former article by myself in the Register, the open condition of roots in young subjects will readily be comprehended. There is no valid reason why a portion of a pulp may not reorganize with a new membrane formed over the part from which the slough took place, though no nerve fibril pass out of such new formed membranal surface.

